# Video game addiction in psychiatric adolescent population: A hospital‐based study on the role of individualism from South China

**DOI:** 10.1002/brb3.3119

**Published:** 2023-06-16

**Authors:** Rui Zhou, Xing‐Yu Xiao, Wen‐Jun Huang, Fei Wang, Xiao‐Qing Shen, Fu‐Jun Jia, Cai‐Lan Hou

**Affiliations:** ^1^ The Second School of Clinical Medicine Southern Medical University Guangzhou China; ^2^ Guangdong Mental Health Center, Guangdong Provincial People's Hospital (Guangdong Academy of Medical Sciences) Southern Medical University Guangzhou China; ^3^ The Division of Psychology Sino‐Singapore Guangzhou Knowledge City Hospital (Guangzhou Huangpu Xinlong Town Central Hospital) Guangzhou China

**Keywords:** childhood trauma, homosexuality, individualism, social media addiction, video game addiction

## Abstract

**Background:**

For decades, video game‐related behaviors have been investigated in different psychologic research, much of whose attention has been paid to video game addiction (VGA), while the differences between VGA and social media addiction (SMA) should have deserved more attention. In addition to detecting common risk factors of VGA, one core question is whether social inclination (individualism or collectivism) matters.

**Object:**

The objectives of this study were to clarify the prevalence of VGA and SMA,, identify the influencing factors of VGA, and clarify the relationships between VGA and adolescents’ individualism–collectivism inclination.

**Method:**

The survey was conducted among 110 adolescent psychiatric patients. For each interviewee, psychological scales were filled face to face. Path analysis was used to examine the causation structure of the childhood trauma‐related symptoms.

**Result:**

The prevalence of VGA was 40.9% (45 out of 110), and it was 41.8% for SMA (46 out of 110); childhood trauma, social media addiction, the individualistic inclination, and the rate of homosexuality were observed to be independent indicators for video game addiction (*r* ^2^ = 0.46).

**Conclusion:**

Psychological counseling on patients’ internet‐related behaviors may focus on the individualistic personality and possible childhood trauma, which are two important risk factors of video game addiction. It is recommended to distinguish between video game addiction and social addiction in clinical practice.

## Graphical abstract

This is the first study that focuses on the relationship between individualistic orientation and video game addiction in China, where the collectivistic culture is dominated.We also discovered that the prevalence for video game addiction is rather high in psychiatric adolescent population; childhood trauma and the individualistic inclination were the independent indicators for video game addiction; homosexuality is negatively associated with video game addiction.

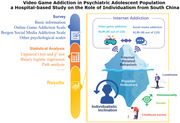

## 1 INTRODUCTION

Video games have quickly become a popular global cultural phenomenon since the turn of the century, drawing lots of players and viewers who identify as young people (Chen et al., 2018). Under the effect of multiple factors, this cultural form not only changes computers and phones into e‐sport platforms (Li, 2022) but also reshapes teens’ behaviors from time to time (Chen et al., 2018; Klimczuk, 2008).

For decades, video game‐related behaviors have been investigated in different psychologic research, with a lot of emphasis on relevant behavioral addictions, as well as the psychological morbidity and difficulties these behaviors cause (Rho et al., 2017; Tonioni et al., 2012).

Leaving aside the aforementioned concerns, video game addiction (VGA) still lacks standard criteria and uniformity scales, causing its description to vary across studies and leaving terminological and conceptual ambiguities from one study to another, despite the World Health Organization's inclusion of “gaming disorder, predominantly online” as a mental disorder in the new International Classification of Diseases with its recommended definition attached (Aarseth et al., 2017). In addition, some clinical literatures may overly generalize online behaviors with the term “Internet addiction” (Starcevic, 2013), which, as far as we know, can be characterized into at least two major types: VGA and SMA (social media addiction) (S.‐Y. Yang et al., 2022).

Yet, what is less uncertain is that the VGA prevalence is rather high in many countries and its clinical consequences are worrisome (Gentile et al., 2017; Kawabe et al., 2020; Stevens et al., 2021) and that several studies on the characteristics of VGA adolescents have revealed that VGA can be caused by a variety of personal, social, and psychological factors (Wu et al., 2013).

Specially, besides aiming at detailing the hospital‐based prevalence and the influential factors of VGA, this study targeted one sociologic trait of VGA adolescents: their individuality–collectivity inclination.

The internet creates a virtual society out of the offline reality, with information and ideas serving as its building blocks (Klimczuk, 2008). In this society, pleasure is more easily, individually, and freely attained than it is in reality. One of the key questions surrounding this phenomenon is whether social orientation (individualism or collectivism) influences video game‐related behaviors. Some limited studies have been conducted on this issue, claiming that individualistic personality may give rise to VGA, but evidence provided was inconclusive (Huang, 2008; W.‐J. Jiang et al., 2018; Stavropoulos et al., 2021).

Studying the connection between social inclination and VGA is important because, as was mentioned, video games have sparked a cultural phenomenon that has a profound impact on adolescent behavior and will gradually modify the entire cultural landscape (this impact may be amplified by the emergence of wearable electronic devices like virtual reality glasses and the Metaverse) (Seddon, 2017; Zarglayoun et al., 2022). Namely, the study of social inclination of VGA adolescents’ personality is not only an investigation to their current psychological condition but also an approach to what kind of cultural and psychological characteristics will there be in their future along with the rapid development of information technology.

Therefore, the purposes of this hospital‐based study were to clarify the prevalence of VGA and SMA (Schou Andreassen et al., 2016), identify the influencing factors of VGA, and clarify the relationships between VGA and adolescents’ individualism–collectivism inclination.

## 2 METHOD

### 2.1 Procedure

The researchers were trained to conduct a series of in‐person interviews. The survey was created specifically for adolescent psychiatric inpatients aged 12–18, with average intelligence. Prior to the survey's launch, all involved researchers had received training on how to properly administer scales and questionnaires, as well as on interpersonal communication. From December 2021 to July 2022, all data were collected randomly in the Mental Health Center of Guangdong Provincial People's Hospital. All interviews were conducted anonymously, and all volunteering respondents were informed of the survey's purpose and content before they provided their written informed consent.

During the survey, no experiential interventions were implemented. We did not alter the patient's medication regimen during data collection; we only asked questions and recorded what is mentioned below. If we found a situation during the interview where a patient did require more care, we would send reminders to his or her supervising doctors.

Clinical Research Ethics Committee of Guangdong Provincial People's Hospital, P. R. China, approved the study protocol. The protocol's registration number is GDREC2018470H (R1), and its approval date is April 29, 2019.

### 2.2 Measures and instruments

#### 2.2.1 Basic sociodemographic information

The survey included several questions concerning gender, age, educational years, sex‐orientation, and so forth, to obtain a profile of the respondent's demographic features.

#### 2.2.2 Diagnostic and medication information

Basic clinical information was collected by referring to their medical records during their hospitalization. Comprehensive diagnostic information came from a structured diagnostic tool, the Mini‐International Neuropsychiatric Interview (MINI). We chose the Chinese version of the MINI as the diagnosis accordance of the respondents (Si et al., 2009). The validity and reliability of the MINI were within acceptable ranges (Sheehan et al., 2010). Medication use was collected, and doses of psychotic drugs were converted into the prescribed daily dose/the defined daily dose ratio (PDD/DDD ratio) (see Table 1).

**TABLE 1:** Comparison of sociodemographic and clinical characteristics between the “ video game addicted” and “not video game addicted” psychiatric adolescent patients.

Variable	Video game addicted (*n* = 45)	Not video game addicted (*n* = 65)	*χ* ^2^ ^a^	df	Sig. ^b^
Male	25 (0.56)	15 (0.23)	12.1	1	**<.01**
Residing in city	37 (0.82)	54 (0.83)	0.0	1	.91
Good academic performance	12 (1.09)	24 (1.04)	6.1	3	.11
Living in school	25 (0.6)	24 (0.37)	5.3	1	**.02**
Authoritative parenting style	8 (0.23)	4 (0.17)	7.6	3	**.05**
Poor parents’ relationship	10 (0.23)	10 (0.15)	2.1	2	.35
Divorced parents	7 (0.16)	5 (0.08)	2.2	3	.53
Living with parents	27 (0.61)	52 (0.8)	9.1	3	**.03**
Psychiatric family history	10 (0.22)	10 (0.15)	0.8	1	.36
Only child of the family	14 (0.33)	15 (0.23)	1.2	1	.28
In a relationship	7 (0.16)	11 (0.17)	3.3	3	.34
Depression (current)	41 (0.91)	46 (0.71)	6.7	1	**.01**
Dysthymia (current)	0.0	9 (0.14)	6.8	1	**.01**
Variable	Video game addicted	Not video game addicted	*t* ^a^	df	Sig.
Age (years)	14.8 ± 1.7	14.8 ± 2	0.1	108	.95
Body Mass Index (kg/m^2^)	21.1 ± 4.2	20.2 ± 3.7	−1.2	101	.24
Homosexuality rate	2.5 ± 1.2	3.3 ± 1.2	3.2	107	**<.01**
Heterosexuality rate	3.2 ± 1.2	3.3 ± 1.2	0.2	107	.82
Education years	8.8 ± 1.5	9.1 ± 1.8	1.0	107	.33
Disease duration (year)	2.4 ± 1.5	2.6 ± 2.1	0.6	107	.53
SGA PDD/DDD	0.7 ± 1.5	2.3 ± 12.0	−0.9	108	.34
Antidepressant PDD/DDD	1.5 ± 1.6	1.2 ± 1.4	−0.9	108	0.37
Benzodiazepine PDD/DDD	0.6 ± 0.7	0.6 ± 0.6	−0.3	108	.73
Total mood‐stabilizers types	0.7 ± 0.6	0.6 ± 0.8	0.7	108	.45
Bergen Social Media Addiction Scale	22 ± 8.4	17.8 ± 8.5	−2.6	108	**.01**
Total ANBFAS score	90.2 ± 61.2	74.5 ± 51.2	−1.5	108	.15
Total CTQ score	57.1 ± 16.7	45.5 ± 15.5	−3.7	106	**<.01**
Total OBVQ score	13.7 ± 6.7	11.6 ± 6.5	−1.6	106	.11
Self‐rating Anxiety Scale	51.2 ± 12.2	45.8 ± 12.5	−2.2	108	**.03**
Self‐Rating Depression Scale	54.6 ± 8.3	51 ± 9.1	−2.1	108	**.04**
Total UCLA Loneliness Scale score	62.1 ± 9.9	56.8 ± 10.8	−2.6	104	**.01**
Total individualism score	76.5 ± 16.1	70.2 ± 20.7	−1.6	98	.11
Total collectivism score	72.8 ± 21.2	77.8 ± 21.8	1.1	98	.26
Individualism minus collectivism	3.7 ± 24.7	−7.6 ± 26.5	−2.1	98	**.04**

*Note*: The categorical variables were expressed as “frequency (proportion)”; continuous variants were presented as “mean ± SD.” Abbreviations: ANBFAS, Adolescent Non‐Suicidal‐Self‐Injury Behavior Function Assessment Scale; CTQ, Childhood Trauma Questionnaire; MDQ, Mood Disorder Questionnaire; NSSI, non‐suicidal self‐ injure; OBVQ, Olweus Bullying/Victimization Questionnaire; PDD/DDD, the quotient of “prescribed daily dose/defined daily dose” of one drug; SGA, second‐generation antipsychotic; Sig., significance.

^a^ Pearson chi‐square test or independent‐samples *t* test.

^b^ Bold values: *p* < .05.

#### 2.2.3 Online game addiction scale

Participants’ video game usage was the main concern of this study. We measured the characteristic by using the Online Game Addiction Scale. It is a newly developed questionnaire in China that contains 13 items reflecting core addiction elements, it is answered on a five‐point Likert scale ranging from 0 (never) to 4 (always), its total score ranges from 0 to 52, and its reliability is 0.971 in the Chinese population (Ma & Dai, 2011). In this study, we regarded a score of no less than 20 as the cut‐off point for VGA, and participants with scores greater than 20 were considered to have VGA. The respondents were accordingly divided into two video game player groups, where the respondents rated no more than 19 were plotted into the “no video game addiction” group, while respondents with GAS scores between 20 and 53 were regarded as the group of “video game addiction.”

#### 2.2.4 Bergen Social Media Addiction Scale

In this study, the social media use was clearly identified as a different internet‐related behavior from video game playing (S.‐Y. Yang et al., 2022). Thereinto, SMA refers to the excessive use of social media like Facebook and Ticktock or watching online porn videos, without involving video games playing. In order to assess SMA, Bergen Social Media Addiction Scale (BSMAS) was used in this study. It consists of six items and queries about participants’ social media use experiences (e.g., “How often did you feel an urge to use social media more and more?”). A seven‐point Likert scale is used, ranging from 1 (very rarely) to 7 (very often) (Love et al., 2015). The Chinese version of the BSMAS was proved to be of good validation and reliability (Russell, 1996). The Cronbach's *α* is .819 in Hongkong, China (Yam et al., 2019).

#### 2.2.5 Adolescent Non‐Suicidal‐Self‐Injury Behavior Function Assessment Scale

Adolescent Non‐Suicidal‐Self‐Injury Behavior Function Assessment Scale (ANBFAS) is a self‐reported 18‐item scale used for measuring the frequency and severity of some common non‐suicidal‐self‐injury behaviors (NSSI) in one's past months (e.g., “intentionally burning or scalding the skin with cigarette butts, lighters or other instruments”) (Feng & Jiang, 2009). Each of the 18 items contains two dichotomies that assess the frequency and severity of one certain NSSI behavior, and the scores of these two parts make up the total score of the scale (Feng & Jiang, 2009).

#### 2.2.6 Childhood Trauma Questionnaire

The questionnaire was used to query about participants’ childhood trauma experiences, consisting of 28 items with a Likert five‐point scale, which comprised five subscales: emotional abuse, emotional neglect, physical abuse, physical neglect, and sexual abuse. The Chinese version of Childhood Trauma Questionnaire (CTQ) showed good reliability and validity in the adolescent population (W.‐J. Jiang et al., 2018; Wang et al., 2018). The scale showed good internal consistency both in non‐clinical samples (Cronbach's *α* = .85) and MDD samples (Cronbach's *α* = .86) (Wang et al., 2022).

#### 2.2.7 Olweus Bullying/Victimization Questionnaire

A modified Chinese version of the Olweus Bullying/Victimization Questionnaire (OBVQ) was used to assess potential peer youth bullying (W. Zhang et al., 1999). In this study, the “bullied” section of the questionnaire was specifically extracted in order to highlight the occurrence of many typical forms of peer bullying. It is rated on a five‐point scale, with “no bullying” being the lowest score and “multiple occurrences per week” being the highest. The Cronbach *α* of the scale in one study on Chinese adolescents was .78 (Y. Yang et al., 2021).

#### 2.2.8 Zung's Self‐rating Anxiety Scale and Zung's Self‐rating depression Scale

Self‐rating Anxiety Scale (SAS) and Self‐rating Depression Scale (SDS) are norm‐referenced scales that are widely used as screeners for anxiety and depression in the departments of psychiatry (Dunstan & Scott, 2020; Zung, 1965). Both scales consisted of 20 items, each of whom scored from 1 to 4 points, resulting in a 20–80 raw score. The scales showed good reliability and validity (Duan & Sheng, 2012; Dunstan et al., 2017). The split‐half reliability of SDS ranged from 0.68 to 0.81; the Cronbach's *α* for SAS is .82 (Dunstan et al., 2017).

#### 2.2.9 UCLA loneliness scale

Twenty‐item UCLA loneliness scale is a broadly used unidimensional measuring instrument for loneliness (Austin, 1983). The UCLA loneliness scale is scored with a four‐point Likert scale, with total scores ranging from 20 to 80. A higher score indicates higher levels of loneliness. Good reliability and validity of the scale were proved, with its coefficient *α* ranging from .89 to .94 (Russell, 1996).

#### 2.2.10 Individualism‐Collectivism Scale

Individualism‐collectivism values were evaluated with the Individualism‐Collectivism Scale (ICS). As indicated by its name, it is combined with two scales assessing the rate of individualism and collectivism, respectively. Specifically, the ICS consists of 27 items, with 13 items measuring individualism (e.g., Being a unique individual is important to me) and 14 measuring collectivism (e.g., It is important to me to maintain harmony in my group). Each of the 27 items is queried on a nine‐point scale ranging from 1 (never) to 9 (always) (Huang et al., 2014). The two dimensions are scored by summing their respective item scores. Fine reliability analyses were also conducted for the ICS and its Cronbach's *α* ranged from 0.64 to 0.83 (Huang et al., 2014). We used the individualism scale score reduced by the collectivism scale score as a criterion for individualistic inclination in this study.

### 2.3 Statistical analysis

SPSS 26.0 (including SPSS Amos 26.0) was used for all statistical analyses. Unpaired *t* test and *χ* ^2^ test were conducted to compare the distribution of each continuous or categorical variable between the “video game addiction” and “no video game addiction” groups; binary logistic regression was performed for proper variances to detect the independent influencing factors for “video game addiction.” Thereafter, proper psychological factors associated to VGA and SMA were included in structural equation modeling (path analysis) via SPSS Amos 26.0, with their good‐of‐fit indexes calculated (Du et al., 2021). Here, due to the lack of normality, the model's indirect effect distributions were calculated with their standard errors generated by bootstrapping (Imai et al., 2010). A two‐sided *p*‐value <.05 was considered statistically significant in the study.

**FIGURE 1:**
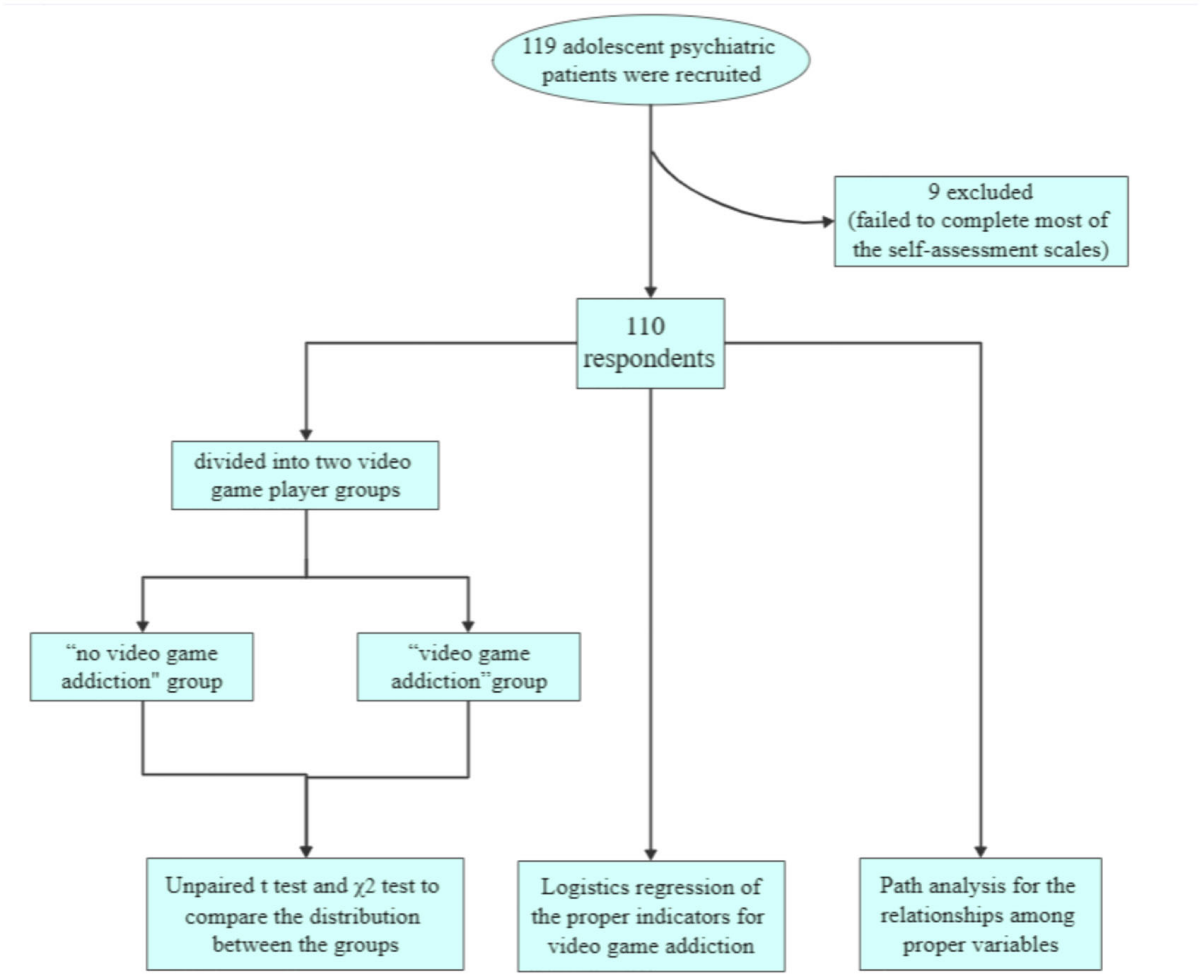
The flowchart of this study.

The flowchart in Figure 1 briefly illustrates the above description.

## 3 RESULT

**FIGURE 2:**
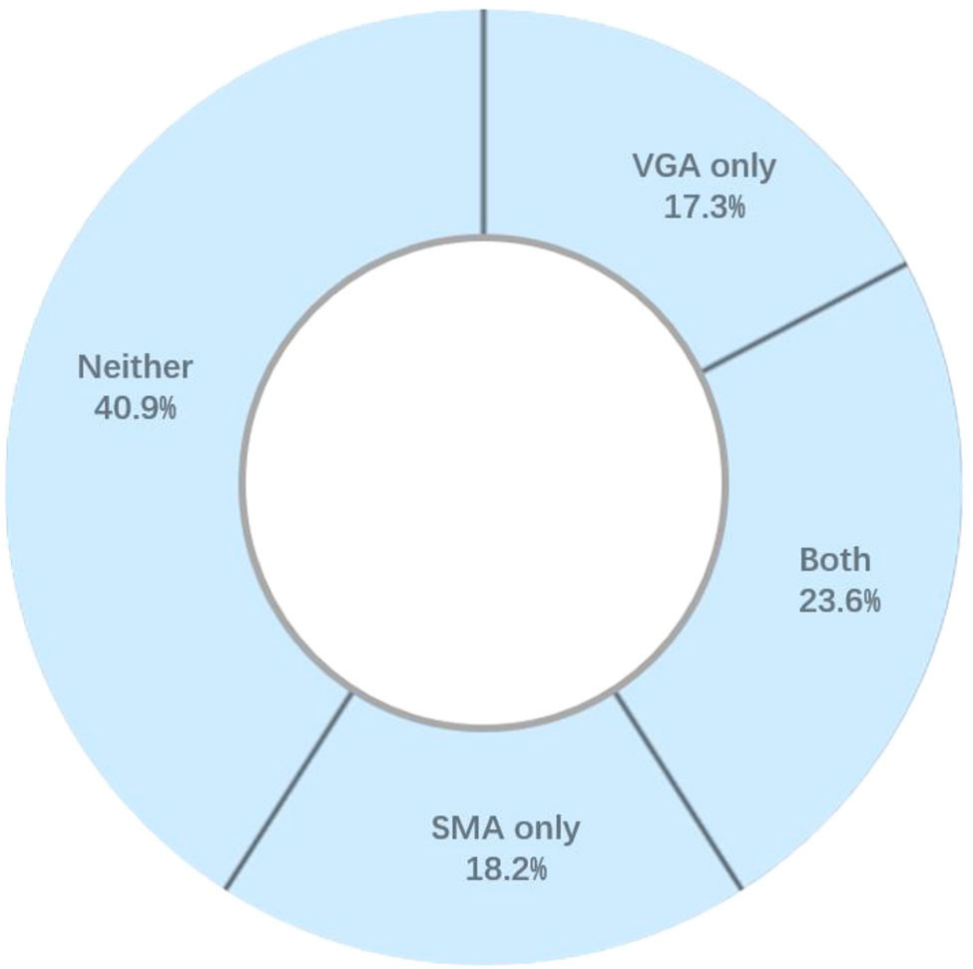
The prevalence of internet addiction. SMA: social media addiction; VGA, video game addiction.

A total of 119 adolescent psychiatric patients were recruited, of which nine failed to complete most of the self‐reported scales; thus, 110 completed the interview, which was conducted in the following analysis. The response rate was 92%. Among them, the prevalence of VGA was 40.9% (45 out of 110), it was 41.8% for SMA (46 out of 110), and it was 23.6% for both VGA and SMA (26 out of 110) (see Figure 2). The mean age was 14.9 years (SD = 1.8 years; range 12–18 years); the mean years of education were 9.0 (SD = 1.7 years; range 5–13 years); 36.4% of them were male; and 96.3% of them were of Han nationality.

Table 1 presents the comparison of sociodemographic and clinical characteristics between the “video game addiction” and “no video game addiction” groups, including social‐demographic information, drugs used, and scores of the scales mentioned above. As for diagnosis information, depression (current) and dysthymia (current) showed significant differences between the two groups, while the other MINI diagnoses observed in this study showed no significant difference.

Table 2 shows the results of binary logistics regression for predicting “video game addiction”; CTQ‐physical neglect score, BSMAS score, the individualistic inclination, and the rate of homosexuality were observed to be independent indicators for VGA (see Table 2, *R* ^2^ = 0.46).

**TABLE 2:** Logistics regression of the proper indicators for video game addiction (*R* ^2^ = 0.46).

Variable	*B*	Wald	df	Sig.^a^	OR	95% CI	
						Lower	Upper
Constance	−2.4	4.8	1	.03			
CTQ‐physical neglect score	0.3	11.5	1	<.01	1.30	1.12	1.52
Bergen Social Media Addiction Scale	0.1	7.0	1	<.01	1.09	1.02	1.17
Individualism minus collectivism	0.0	4.3	1	.04	1.02	1.00	1.05
Rate of homosexuality	−0.8	12.1	1	<.01	0.43	0.27	0.69

Abbreviation: CI, confidence interval; CTQ, Childhood Trauma Questionnaire; Sig., significance.

^a^ *p* < .05.

**FIGURE 3:**
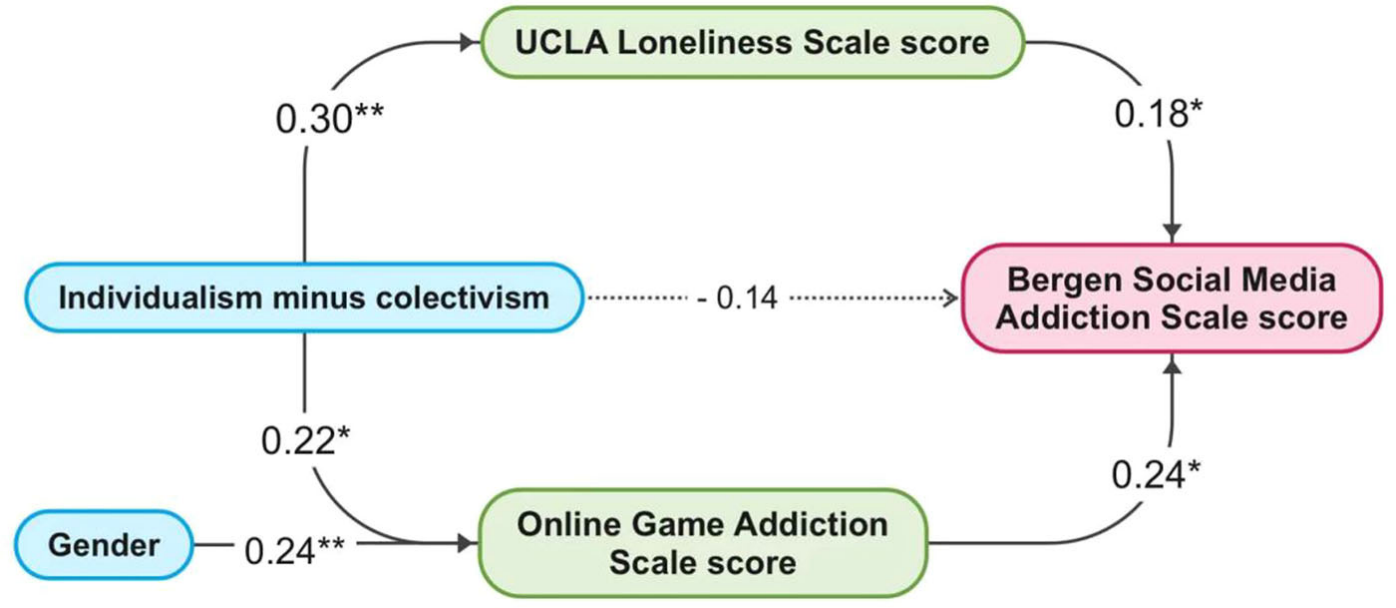
The causations among proper variables. Lines with arrows indicate causations; each number on every line makes the standardized regression weight of every two variances linked; blue bars indicate the independent variable (or exogenous variable); green bars indicate the intermediate variables (or endogenous variables); red bars indicate dependent variables; *p < .05, **p < .01; the dash line means the path shows no significance.

Proper variables were subjected to path analysis, and the detailed information including the direct and indirect effects of the involved paths are shown in Table 3. A comprehensive causation network of proper variables is shown in Figure 3. The individualistic inclination (individualism minus collectivism) is directly linked with OGDQ score and loneliness score, and indirectly with BSMAS. The fit indexes of the path analysis model are shown in Table 4, most of which were within their reference ranges, indicating that the model is fit.

**TABLE 3:** Path analysis for the relationships among proper variables.

Direct causation			Estimate^a^	SE	CR	*p* ^b^
Individualism minus collectivism	→	Gaming addiction scale score	0.22	0.03	2.44	**.02**
Individualism minus collectivism	→	Loneliness scale score	0.30	0.04	3.39	**<.01**
Gender	→	Gaming addiction scale score	0.24	1.68	3.38	**<.01**
Loneliness scale score	→	BSMAS	0.18	0.08	2.01	**.04**
Gaming addiction scale score	→	BSMAS	0.24	0.09	2.63	**0.01**
Individualism minus collectivism	→	BSMAS	‐0.14	0.03	‐1.62	.11
Indirect causation			Estimate^a^	95% CI		*p*
				Lower	Upper	
Individualism minus collectivism	→	BSMAS	–	0.01	0.07	**.04**

Abbreviation: BSMAS, Bergen Social Media Addiction Scale.

^a^ Linear regression analysis (path analysis). The estimates show the standardized regression weights of the paths.

^b^ Bold value: *p* ≤ .05.

**TABLE 4:** Fit indexes of the path analysis.

Fit index	Value	Reference range^a^
*χ* ^2^/df	1.75	< 3
CFI	0.93	>0.9
NFI	0.88	>0.9
RMSEA	0.08	<0.1

Abbreviations: CFI, comparative fit index; NFI, normed fit index; RMSEA, root mean square error of approximation.

^a^ The fit index within its reference range indicates that the path analysis model is fit.

Table 5 shows the correlation analysis among the total scores of the scales showing significant differences between the addicted and non‐addicted groups. The correlation analysis also included all the variables in the path analysis, so as to demonstrate more details of the characteristics of the studied samples.

**TABLE 5:** Correlation analysis among the proper variables (including all the variables in path analysis).

	Variables	1	2	3	4	5	6	7	8
1	GAS	1							
2	Individualism	0.12	1						
3	Collectivism	−0.15	0.17	1					
4	BSMAS	**0.24**	0.11	0.15	1				
5	CTQ	**0.35**	0.18	**−0.26**	**0.28**	1			
6	SAS	0.17	0.13	−0.11	**0.36**	**0.45**	1		
7	SDS	**0.25**	0.04	−0.10	**0.25**	**0.47**	**0.82**	1	
8	MDQ	0.11	0.15	−0.04	**0.20**	**0.24**	**0.36**	**0.35**	1
9	UCLA	0.16	0.12	**−0.28**	0.18	**0.44**	**0.58**	**0.62**	**0.37**

*Note*: The number on the left of each variable refers to the same variable in the longitudinal headings. Individualism and collectivism refer to Individualism–Collectivism Scale. Bold value: *p* < .05.

Abbreviations: BSMAS, Bergen Social Media Addiction Scale; CTQ, Childhood Trauma Questionnaire; GAS, Gaming Addiction Scale Score; MDQ, Mood Disorder Questionnaire; SAS, Zung's Self‐rating Anxiety Scale; SDS, Zung's Self‐rating depression Scale; UCLA, UCLA Loneliness Scale score.

## 4 DISCUSSION

The prevalence of VGA was high in psychiatric adolescent patients. Besides individualistic inclination, childhood trauma and gender were the influential factors of VGA in this study. To our best knowledge, this is the first study that focuses on the relationship between individualistic orientation and VGA in China, where the collectivistic culture is dominated.

Though especially distinguished from social media addiction in this study, the rate of VGA in psychiatric adolescent patient population was higher than that reported in previous studies, where the internet addiction prevalence ranged from 1.5% to 8.2% in Europe and America (Jorgenson et al., 2016; Weinstein & Lejoyeux, 2010), and from 6.7% to 17.9% in Asia (Yen et al., 2007) (without distinction of VGA and SMA).

This discrepancy is likely related to the sampling source and period. On the one hand, unlike community‐based studies, the sample in this study was drawn from a psychiatric adolescent patient population, and it appears that internet addiction is a common co‐morbidity of psychiatric disorders (Shaw & Black, 2008). The data, on the other hand, were gathered during the COVI‐19 pandemic, when the lockdown policy left adolescents at home unprepared, making the internet a major source of communication and entertainment (J. Gao et al., 2020; Ko & Yen, 2020; Meng et al., 2022; Sun et al., 2020). In addition, some of the trans‐study discrepancies may be due to different scales used in assessing internet behaviors (Dahl & Bergmark, 2020; M. W. B. Zhang et al., 2018). Thus, VGA is of interest because of its high prevalence and heterogeneity across time and populations.

As forementioned, internet addiction may relate to a series of psychiatric disorders. Specifically, we detected significant differences of the physical neglect score (CTQ), SAS and SDS between the “video game addiction” and “non‐video game addicted” groups. Further, the CTQ‐physical neglect score was found to be an independent indicator of VGA (see Tables 1 and 2). These findings were consistent with some previous studies, suggesting that childhood trauma may have an impact on VGA via depression and anxiety symptoms, both directly and indirectly (Bickham, 2021; Shokouhi‐Moqhaddam et al., 2013). One generalizable explanation could be that for victims of childhood trauma, video games may be one of the available coping strategies to trauma‐related symptoms (Sheng et al., 2022; Shi et al., 2020).

Furthermore, internet‐addicted adolescents showed more individualism in their personality (both VGA and SMA) (see Table 1 and Figure 3), and interestingly this characteristic was detected only when VGA was distinguished from SMA (S.‐Y. Yang et al., 2022). Thus, we accordingly deduce from Figure 3 that individualism is directly link to VGA and indirectly related to SMA, the latter of which was fully mediated by VGA and loneliness (F. Gao et al., 2018; Q. Jiang et al., 2018; Sönmez et al., 2021). There was no direct relationship between individualism and SMA. Accordingly, it is argued that SMA might be alleviated spontaneously when VGA adolescents have more alternative hobbies instead of video games and engage in more offline social activities to reduce loneliness.

Thereinto, the influence of individualism on how adolescents obtain rewards is explanatory. Individualistic participants’ well‐being may be derived from their independent judgment on personal needs, while collectivism emphasizes the happiness based on the security and stability of interpersonal relationships (Huang, 2008). In this context, it is obvious that unlike social media, video games provide more personalized services for individualistic adolescents while not overestimating their ties to others (Q. Jiang et al., 2018; Stavropoulos et al., 2021).

Another interesting topic is the relationship between VGA and gender characteristics. As we can see in Table 2, as an independent predictor, homosexuality is negatively associated with VGA. Gays may find less identity in gaming because there are more masculine‐ and heterosexual‐oriented norms to follow in video games (Luk et al., 2019). This idea is consistent with another finding of this study: male adolescents exhibited a greater propensity for VGA (see Table 1 and Figure 3) (Stevens et al., 2021).

To sum up, VGA is a complicated syndrome affected by bio‐psycho‐social factors. From a biological point of view, adolescents with VGA are more masculine; from a clinical psychological perspective, they suffer from more depression and anxiety which are probably the emotional continuation of childhood trauma; on a sociological level, they are more individualistic.

### 4.1 Strength and limitation

The first strength of this study was that we built a causation model to clarify the relationships of internet‐related factors, whose good‐of‐fit indexes were proper. The second strength was that it was a hospital‐based study, which was rare in previous research. The third strength was that we have made a comparison between GVA and SMA and detailed many specific characteristics of them, which was rarely done in previous studies (Jaiswal et al., 2020; Marin et al., 2021). Forth, we paid attention to the sex minority and discussed the related findings of them. Fifth, this is the first study that focuses on the direct effect of individualism orientation on VGA in the Chinese adolescent population.

This study had some limitations. One was that the cross‐sectional design of the study limited the exploration of the between‐variable causation, and the conclusion of the study needs more confirmation from longitude studies. The second limitation was that some self‐reported scales used in this study might be biased. The third limitation was that all of our samples were from hospitalized populations, which may also be a source of selection bias.

## 5 CONCLUSION

The prevalence of VGA in teenage psychiatric patients was found to be high in this cross‐sectional study. In clinical practice, it is recommended to distinguish between VGA and social addiction, with the former receiving priority attention; counseling on patients’ internet‐related habits may concentrate on the individualistic personality and possibly childhood trauma, both of which are key risk factors for VGA. Finally, internet addiction was likely to be quite complex, and our findings needed to be validated further by longitude investigations.

## AUTHOR CONTRIBUTIONS

Cai‐Lan Hou and Rui Zhou analyzed and interpreted the patient data. Rui Zhou was the major contributor in writing the manuscript. Cai‐Lan Hou and Fu‐Jun Jia designed the study. Rui Zhou, Xing‐Yu Xiao, Wen‐Jun Huang, Xiao‐Qing Shen, and Fei Wang were responsible for sample collection and psychological evaluation. Rui Zhou participated in statistical analysis of data and prepared the tables and figures. Cai‐Lan Hou made in‐depth revisions to the manuscript. All authors read and approved the final manuscript.

## CONFLICT OF INTEREST STATEMENT

The authors declare no conflict of interest.

### PEER REVIEW

The peer review history for this article is available at https://publons.com/publon/10.1002/brb3.3119

## Data Availability

The datasets analyzed during the current study are available from the corresponding author on reasonable request.
